# Moxibustion for cancer-related fatigue: study protocol for a randomized controlled trial

**DOI:** 10.1186/s12906-017-1856-3

**Published:** 2017-07-05

**Authors:** Mikyung Kim, Jung-Eun Kim, Hye-Yoon Lee, Ae-Ran Kim, Hyo-Ju Park, O-Jin Kwon, Eun-Jung Kim, Yeon-Cheol Park, Byung-Kwan Seo, Jung Hyo Cho, Joo-Hee Kim

**Affiliations:** 1 Clinical Research Division, Korea Institute of Oriental Medicine, 1672, Yuseongdae-ro, Yuseong-gu, Daejeon, 34054 Republic of Korea; 2 College of Korean Medicine, Dongguk University, 123, Dongdae-ro, Gyeongju, Gyeongsangbuk-do 38066 Republic of Korea; 3 Department of Acupuncture & Moxibustion, Kyung Hee University Hospital at Gangdong, 892, Dongnam-ro, Gangdong-gu, Seoul, 05278 Republic of Korea; 4 Department of Internal Medicine, College of Traditional Korean Medicine, Daejeon University, 62, Daehak-ro, Dong-gu, Daejeon, 34520 Republic of Korea

**Keywords:** Cancer-related fatigue, Moxibustion, Randomized controlled trial, Traditional Korean medicine

## Abstract

**Background:**

Cancer-related fatigue is one of the most common symptoms experienced by cancer patients, and it diminishes their quality of life. However, there is currently no confirmed standard treatment for cancer-related fatigue, and thus, many patients who suffer cancer-related fatigue seek complementary and alternative medicines such as moxibustion. Moxibustion is one of the most popular therapies in traditional Korean medicine used to manage fatigue. Recent studies have also demonstrated that moxibustion is effective for treating chronic fatigue. However, there is insufficient evidence supporting the effect of moxibustion against cancer-related fatigue. The aim of this study is to assess the efficacy and safety of moxibustion treatment for cancer-related fatigue.

**Methods/design:**

A multi-center, three-armed parallel, randomized controlled trial will be conducted. Ninety-six patients with cancer-related fatigue will be recruited from three clinical research centers. They will be randomly allocated to one of three groups in a 1:1:1 ratio. The moxibustion group will receive moxibustion treatment at CV8, CV12, LI4 and ST36. The sham moxibustion group will receive sham moxibustion at non-acupoints. Both the moxibustion and sham moxibustion groups will receive 30-min treatments twice a week for 8 weeks. The usual care group will not receive moxibustion treatment. All participants will be educated via a brochure on how to manage cancer-related fatigue in daily life. The outcome measurements will be evaluated at baseline, week 5, week 9, and week 13 by assessors who are blinded to the group allocation. The primary outcome measure will be the mean change in the average scores of the Brief Fatigue Inventory before and after treatments between groups. The secondary outcome measures will be the mean difference in changes from baseline of the Brief Fatigue Inventory, functional assessments of cancer therapy-fatigue, European Organization for Research and Treatment of Cancer Quality of Life Questionnaire C-30 scores, and Montreal Cognitive Assessment scores between groups. Safety will be assessed by monitoring adverse events at each visit.

**Discussion:**

The results of this study will provide evidence to confirm whether moxibustion can be used as a therapeutic option for treating cancer-related fatigue.

**Trial registration:**

Clinical Research Information Service KCT0002170. Registered 16 December 2016.

**Electronic supplementary material:**

The online version of this article (doi:10.1186/s12906-017-1856-3) contains supplementary material, which is available to authorized users.

## Background

As the prevalence of cancer and the survival rate of patients increase, the quality of life (QOL) of cancer survivors and the symptoms affecting QOL are gaining increased attention [1]. Cancer-related fatigue is one of the most common symptoms that cancer patients experience [2] and is among the most prominent residual or persistent symptoms of cancer survivors [3]. Cancer-related fatigue often occurs during the cancer treatment period, although it may occur independent of treatment and sometimes continues to persist even after the end of treatment or the complete remission of cancer [4].

Cancer-related fatigue reduces the level of energy and decreases the QOL of cancer patients, negatively affecting the return-to-work rate of cancer survivors [5, 6]. It also seriously damages the QOL of the families in addition to cancer patients in terms of physical, social, economic, psychological and spiritual aspects [7]. However, it is not a fatal condition that directly threatens the survival of cancer patients. Therefore, it is recognized to be relatively less important than other critical issues regarding cancer [8]. Moreover, the definition of cancer-related fatigue is completely dependent on the subjective sense of tiredness felt by the patient, and therefore, it is easy to overlook when the patient does not actively report the symptom [8].

The mechanism of cancer-related fatigue has been reported to be associated with cytokine production or mediators of inflammatory responses of cancer and/or cancer treatment [9]. In particular, tumor necrosis factor (TNF) alpha signaling seems to be closely related to cancer-related fatigue [6]. In addition to inflammation and latent viral reactivation [10, 11], other various factors such as oxidative stress [12], changes in the neuroendocrine system including dysregulation of the hypothalamic-pituitary-adrenal (HPA) axis, autonomic nervous system disorders including central vagal stimulation, serotonin dysregulation [10, 11], and changes in muscle and ATP metabolism [11], have been reported to be involved in cancer-related fatigue. However, the mechanism of cancer-related fatigue has not yet been fully elucidated [13].

There are various known risk factors related to cancer-related fatigue, including the stage of cancer, the specific type of cancer (breast, colon, liver) and diabetes, high body mass index, genetic factor, psychological and biobehavioral factors such as the level of fatigue before cancer treatment, depression, sleep disturbances, physical activity and physical deconditioning, negative expectation, etc. [10, 14, 15]. The cancer itself, various cancer treatments, and accompanying diseases such as anemia, cachexia, etc., also appear to be related to cancer-related fatigue, but the degree of their contributions and how such risk factors interact remain unclear [11]. To date, the cause of cancer-related fatigue has not been explicitly identified [11, 14].

The first step in managing cancer-related fatigue is determining the possible causes of fatigue and correcting them [4, 8, 16]. However, as previously discussed, a variety of factors are involved and contribute to cancer-related fatigue, and therefore, it is very difficult to identify specific causes and to completely eliminate fatigue among cancer patients [14]. There is currently no standard therapy for treating cancer-related fatigue [6, 17].

For pharmacologic therapy to mitigate cancer-related fatigue, antidepressants and psychostimulants such as methylphenidate, modafinil, and bupropion and steroids such as dexamethasone have been proposed and studied, but the results of clinical trials are inconsistent [4, 16–18]. According to a recent meta-analysis, methylphenidate was significantly better than placebo, while the effect of modafinil was similar to that of placebo, although further studies are needed to confirm this conclusion [17]. Considering that the clinical effects of these drugs are unclear and the risk of adverse drug reactions are of high concern, especially for long-term use, no specific drug is currently recommended as a primary therapeutic option for cancer-related fatigue [17, 19, 20].

Despite the lack of a standard therapy for cancer-related fatigue, the high number of patients who suffer from cancer-related fatigue has created a demand for complementary alternative medicine (CAM). A systematic review reported that CAM utilization rates among cancer patients range from 11 to 95% [21]. According to a survey targeting breast cancer survivors in the United States, one third of patients used CAM [22]. Another survey conducted among South Korean cancer patients reported that more than half of respondents used CAM [23].

Moxibustion is one of the most commonly used treatments in traditional medicine in East Asia including Korea. It has been used to prevent and treat various diseases by warming the body and promoting the energy of patients [24, 25]. Moxibustion is particularly good at supplementing energy (*qi*), and thus, it has been often applied to patients who experience fatigue or similar conditions [25]. Modern scientific studies also have shown that moxibustion is effective in alleviating chronic fatigue [26–28], and some parts of its mechanism have been reported to be related to activation of the vagus nerve [26], reduction of oxidative damage [26, 27, 29], improvement of inflammation [30], regulation of the HPA axis [10], and upregulation of hippocampal progranulin [10].

Lee et al. [31] showed the possibility of using moxibustion to treat cancer-related fatigue through a systematic review and meta-analysis of related literature. However, all the studies included in this review were reported and conducted only in China, and most of the studies had high risk of bias. The details of moxibustion treatment used in each study were not described based on the Consolidated Standards of Reporting Trials (CONSORT) or on the Standards for Reporting Interventions in Clinical Trials of Acupuncture (STRICTA), and most of the studies did not report of adverse events (AEs) and drop-outs. Thus, Lee et al. [31] concluded that a well-designed clinical trial with better reporting quality is warranted to draw any conclusion regarding the effect of moxibustion for treatment of cancer-related fatigue. Recently, Mao et al. [32] demonstrated that their newly devised method of infrared laser moxibustion was more effective in reducing the level of fatigue than sham moxibustion. However, infrared laser moxibustion is not yet commonly used in Korea. More evidence is needed to confirm the effectiveness and safety of moxibustion against cancer-related fatigue.

Therefore, we designed this randomized controlled trial to compare the clinical responses of moxibustion, sham moxibustion, and usual care interventions in patients with cancer-related fatigue.

## Methods/design

### Design

**Fig. 1:**
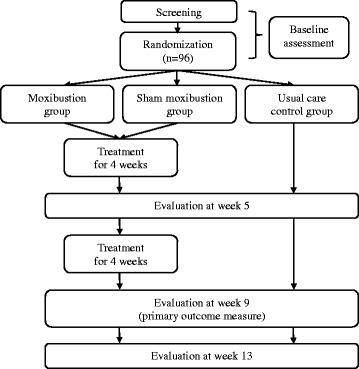
Flow chart of the study

This is a multi-center, three-armed parallel, randomized controlled trial. Ninety-six participants who meet the diagnostic criteria for cancer-related fatigue of the international classification of diseases, tenth revision clinical modification (ICD-10-CM), will be randomly allocated to one of the three groups in a 1:1:1 ratio. One group will be the treatment group (moxibustion group), and the other two will be control groups (sham moxibustion group and usual care group). The flowchart of this trial is shown in Fig. 1.

### Recruitment

Three clinical research centers in South Korea will recruit participants: Daejeon Oriental Hospital of Daejeon University, Kyung Hee University Oriental Hospital at Gangdong, and Dongguk University Bundang Oriental Hospital.

To ensure enough patients, clinical trial information will be posted on notice boards of the hospitals both on and offline. Recruitment notices will also be released on flyers, daily local newspapers, and advertisement boards at public places.

### Eligibility criteria

#### Inclusion criteria

1. Male or female aged 19 or over but under 80 years
2. Cessation of cancer-related treatments (e.g., surgery, chemotherapy, radiotherapy, immunotherapy) at least 12 weeks before the trial (on-going hormone therapy, which must have been initiated at least 3 weeks prior to enrollment, is allowed)
3. Continuous fatigue related to cancer treatment or cancer itself for at least 4 weeks, fulfilment of the ICD-10-CM diagnostic criteria for cancer-related fatigue, and a brief fatigue inventory (BFI) score of 4 or more
4. An Eastern Cooperative Oncology Group (ECOG) performance status ≤2
5. Willingness to participate in the trial and having provided written consent

#### Exclusion criteria

1. Having maintained the current level of fatigue prior to the diagnosis of cancer
2. Severe anemia (platelet count <60,000/μL, hemoglobin <8 g/dL, or absolute neutrophil count <1000/μL)
3. Receiving aggressive treatment for anemia (e.g., erythropoietin or blood transfusion)
4. Poor oral intake with a lower-than-normal level of serum albumin
5. Any significant sign or symptom of inflammation with C-reactive protein (CRP) ≥ 10 mg/L and white blood cell >10,000/μL
6. Abnormal findings in a thyroid function test (abnormal level of free thyroxine and thyroid stimulating hormone <0.1 μIU/ml or TSH > 5.1 μIU/ml)
7. Abnormal findings in a liver function test or a renal function test (aspartate aminotransferase or alanine aminotransferase ≥2× upper normal range or creatinine ≥2.0 mg/dL) or the presence of serious liver failure or renal failure
8. A score of 11 points or more on either the anxiety or depression subscales of the Hospital Anxiety and Depression Scale (HADS)
9. An insomnia severity index (ISI) score of 15 points or more
10. Level of cancer pain measured by the numeric rating scale (NRS) ≥ 4
11. An estimated life expectancy of six months or less
12. A plan of surgery, chemotherapy, or radiotherapy during the study
13. History of medication (methylphenidate, modafinil, bupropion, dexamethasone) to manage cancer-related fatigue taken within 4 weeks of the beginning of the trial
14. History of Korean medical treatment (e.g., acupuncture, moxibustion, cupping, or herbal medicine, etc.) to manage cancer-related fatigue within 4 weeks of the beginning of the trial
15. Initiation or a change in dietary supplement regimen or non-pharmacologic therapies (e.g., cognitive behavioral therapy, exercise, etc.) for alleviating cancer-related fatigue during the trial or within 4 weeks of the beginning of the trial
16. Having participated in other clinical trials within 4 weeks of the beginning of the trial
17. History of hypersensitivity reactions or serious adverse reactions (SAEs) after moxibustion treatment or an inability to cooperate with moxibustion treatment due to other reasons such as dyspnea
18. Women who are pregnant, lactating, or planning to become pregnant
19. Presence of other apparent factors or any diseases that could cause current fatigue other than cancer treatment or the cancer itself

### Randomization and allocation concealment

Random numbers will be generated by SAS (Version 9.4, SAS institute. Inc., Cary, NC) and sealed in sequentially numbered opaque envelopes. The envelopes will be delivered to each research center and kept in a double-locked cabinet. The practitioners of each research center will open the envelops and allocate the participants who meet the eligibility criteria after signing an informed consent to one of the three groups according to the contents of the envelops.

### Blinding

Practitioners cannot be blinded because of the unique nature of moxibustion treatment. Although the subjects of the usual care group will be able to determine which group they are allocated to, the participants in the moxibustion and sham moxibustion groups will be blinded. To assess whether the patient-blinding was successfully achieved among the moxibustion group and sham moxibustion group, the subjects of these two groups will undergo a blinding test and a credibility test after the first and final treatment. Outcomes will be assessed by researchers who are not involved in the intervention procedure. Thus, the assessors will be blinded to all three groups, while the participants will be blinded in only the moxibustion and sham moxibustion groups.

### Intervention

There are three arms in this trial. All participants regardless of their assignment will be educated via a brochure on how to manage daily life to alleviate the cancer-related fatigue.

#### Moxibustion group

**Fig. 2:**
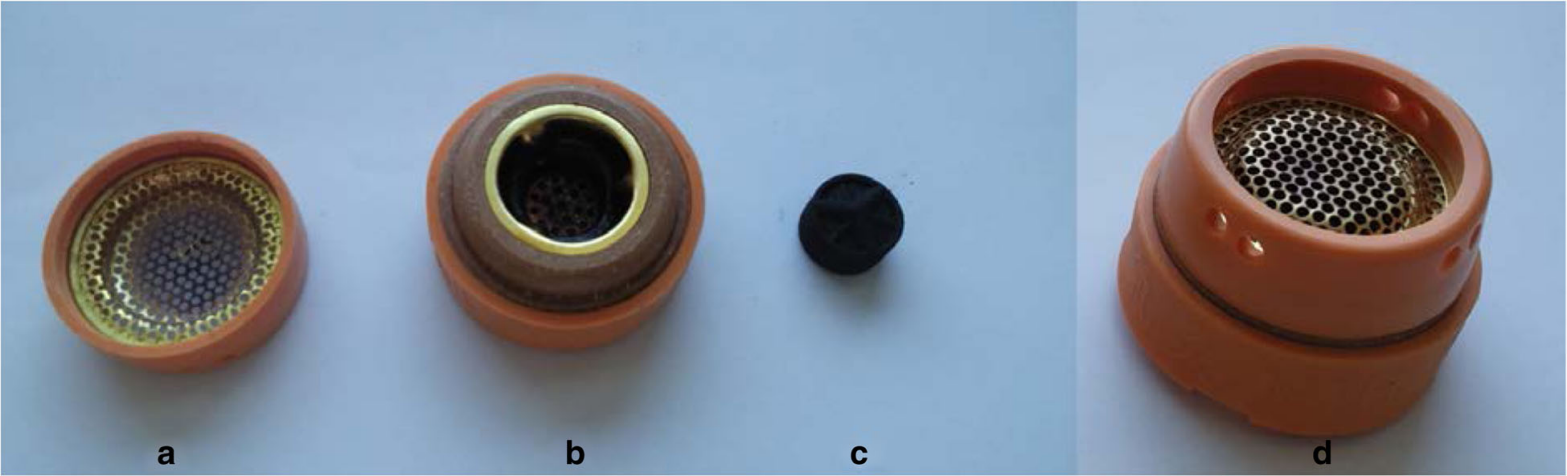
Ignition-type moxibustion device. **a**, safety cap; **b**, body of the device; **c**, mugwort moxa cone; **d**, the assembled device of **a**, **b**, **c** and **d**

**Fig. 3:**
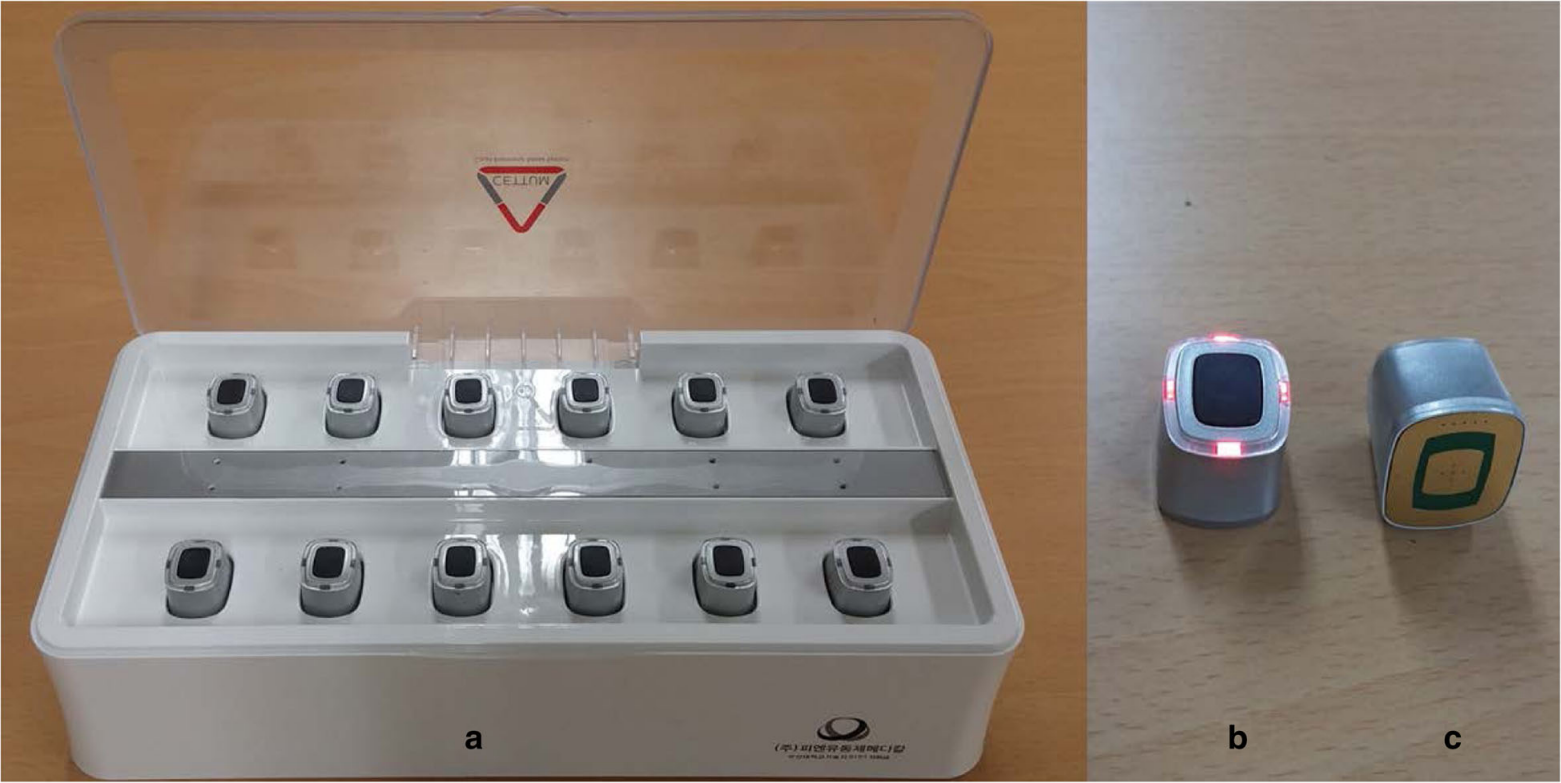
Electrical moxibustion apparatus. **a**, twelve moxibustion devices in a charger; **b**, a powered-up moxibustion device; **c**: the bottom of the moxibustion device embedded with an electrically heating board

Participants in the moxibustion group will receive moxibustion treatment via two types of moxibustion devices: an ignition-type moxibustion device (IM, Fig. 2) and an electrical moxibustion apparatus (EM, Fig. 3).

**Fig. 4:**
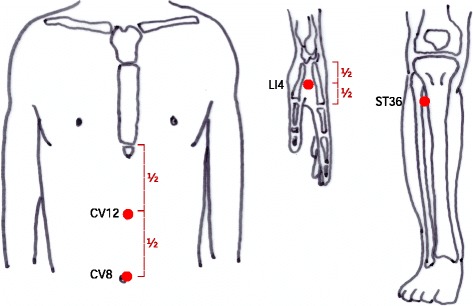
Acupoints for the moxibustion group

The IM is composed of a red clay body covered by a silicon sheath and safety cap (Fig. 2). An ignited mugwort moxa cone will be inserted into the body of the IM. The base of the body has tiny holes that acts as a heat channel. Two IMs will be sequentially applied at two acupoints on the abdomen, CV8 and CV12 (Fig. 4).

The EM is a cube-shaped apparatus with a base embedded in an electrical board controlled by a heat sensor (Fig. 3). The EM can be stably fixed on the body by attaching double-sided medical tape to the base of the EM. Four EMs will be sequentially attached at acupoints on the hands and legs, bilateral LI4 and ST36 (Fig. 4). When the button on the top of the EM is pushed, the board on the base starts to emit heat and the light on the top begins to blink. The plateau temperature of the base of the EM is 45 °C. The treatment time is 30 min, and the participants in this group will receive the treatment twice per week for 8 weeks. Detailed explanations of the moxibustion treatments are tabulated according to the revised STRICTA checklist for moxibustion (Additional file 1).

#### Sham moxibustion group

**Fig. 5:**
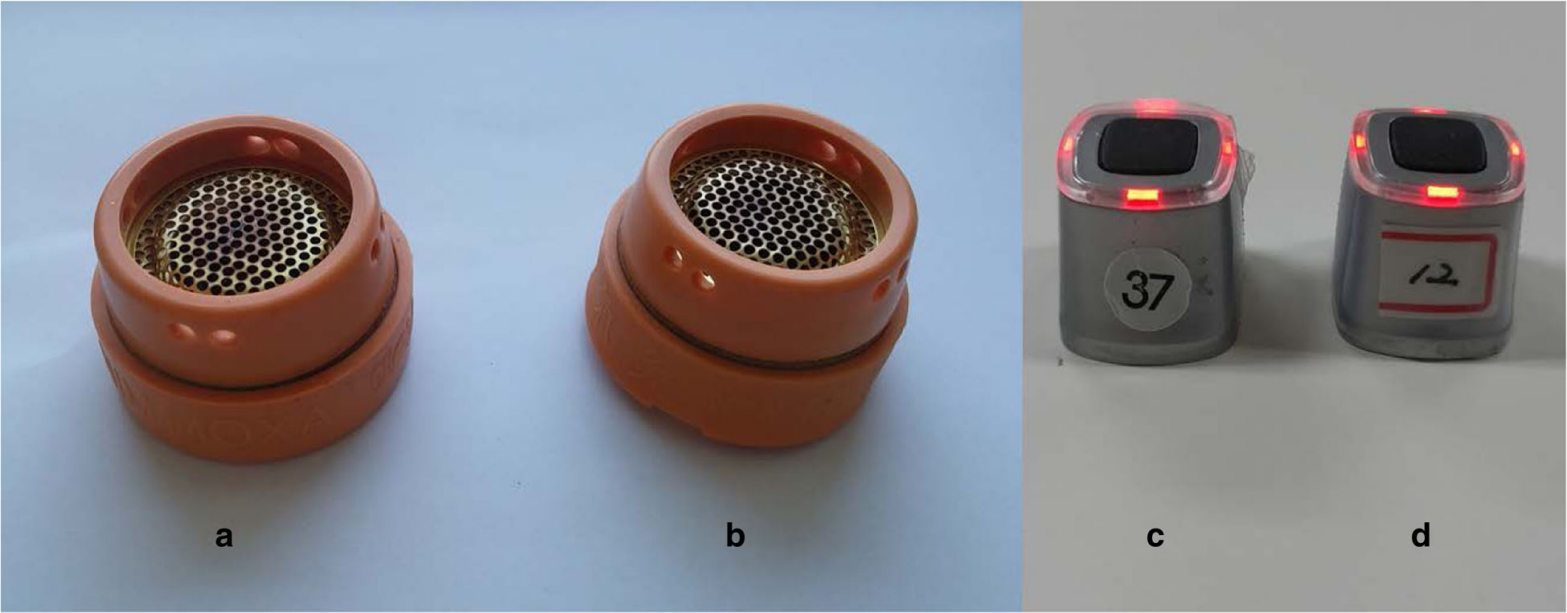
A front view of moxibustion. **a** & **b**, ignition-type moxibustion devices; **a**, real; **b**, sham; **c** & **d**: electrical moxibustion apparatus; **c**, real; **d**, sham. The front views of the real and sham moxibustion devices are identical

The appearances of the IM and EM used for the sham moxibustion group will be exactly identical to those used for the moxibustion group (Fig. 5). The participants will be able to see the practitioner ignite the mugwort and the IM with a small amount of smoke and burning odor applied on their abdomen. In the case of the EM, the participants will be able to see that the EM attached on their skin is turned on as the light on the top of the EM begins to blink. These procedures will be the same as the method used for the moxibustion group.

**Fig. 6:**
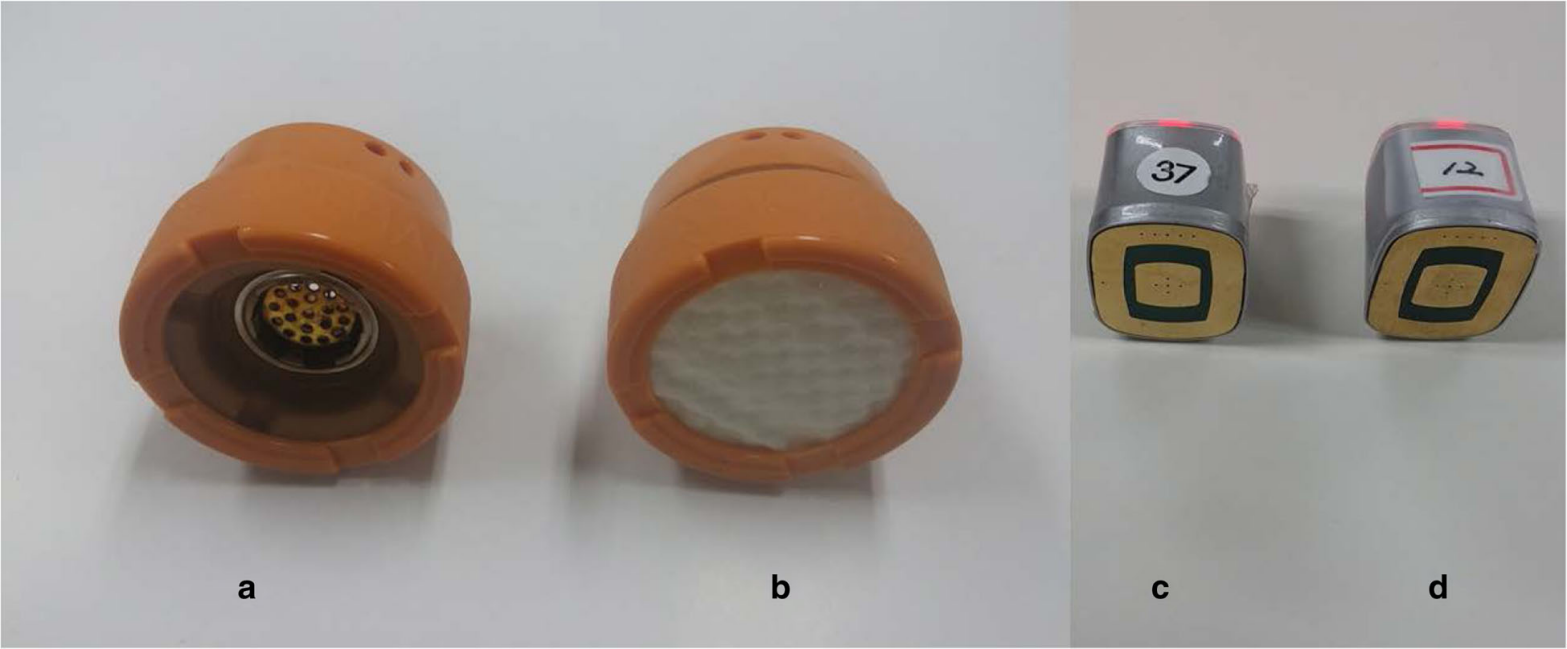
A bottom view of moxibustion. **a** & **b**, ignition-type moxibustion devices; **a**, real; **b**, sham; **c** & **d**: electrical moxibustion apparatus; **c**, real; **d**, sham

**Fig. 7:**
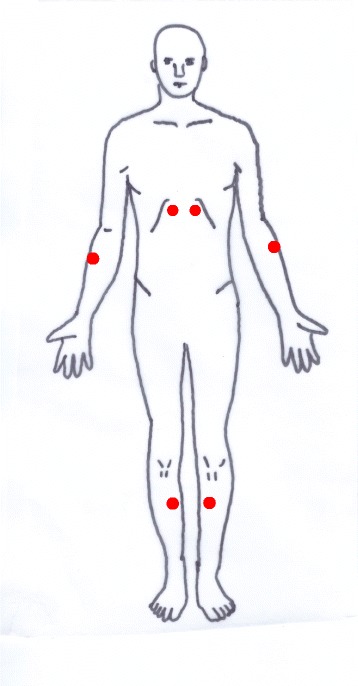
Non-acupoints for the sham moxibustion group

However, the base of the IM will be filled with insulator made of styrofoam, and the heat sensor in the electrical board of the base of the EM will be controlled to block heat transfer (Fig. 6). The sham moxibustion will be applied at non-acupoints that are irrelevant to fatigue. The locations of the points that will be used for this group are as follows (Fig. 7): abdomen (approximately 13.5 cm above the bilateral 3 cm from the umbilicus), upper limb (1 cm lateral and 5 cm distal to the cubital creases of the bilateral arms), and lower limb (upper 1/3 points of the medial line of the bilateral tibia). The sham IMs will be applied on the abdomen points, and the sham EMs will be attached on the upper and lower limb points.

#### Usual care group

The participants in the usual care group will not receive moxibustion treatment. They will maintain their usual treatment and self-care but will not begin any additional treatment to improve their cancer-related fatigue during the study.

### Prohibited and permitted concomitant treatment

The participants will be prohibited from receiving any pharmacologic treatment of Western medicine or any other interventions of traditional Korean medicine to manage cancer-related fatigue other than the interventions provided by the trial. If they continued to receive other nonpharmacologic therapies such as exercise, yoga, meditation, etc. or were already taking any dietary supplements to mitigate cancer-related fatigue at least 4 weeks before the trial was initiated, they will be able to maintain the therapies. However, any treatment used to attenuate cancer-related fatigue cannot be newly introduced after the initiation of the trial. All new treatments begun after the beginning of the trial and concomitant medications to treat medical conditions unrelated to cancer-related fatigue will be recorded on the case report form.

### Study schedule of enrolment, allocation, and assessments

After eligibility screening of the candidate participants who had signed informed consent, the participants will be randomly allocated to one of the three groups. Before the initial intervention begins, baseline measures will be obtained. After four weeks of intervention, outcomes reflecting the level of fatigue will be assessed at week 5. Post-treatment assessments will be conducted at week 9 after completing eight weeks of intervention. The follow-up visit will be planned at week 13, concluding the trial. The schedule of the enrolment, interventions, and assessments is given in Table 1.

**Table 1:**
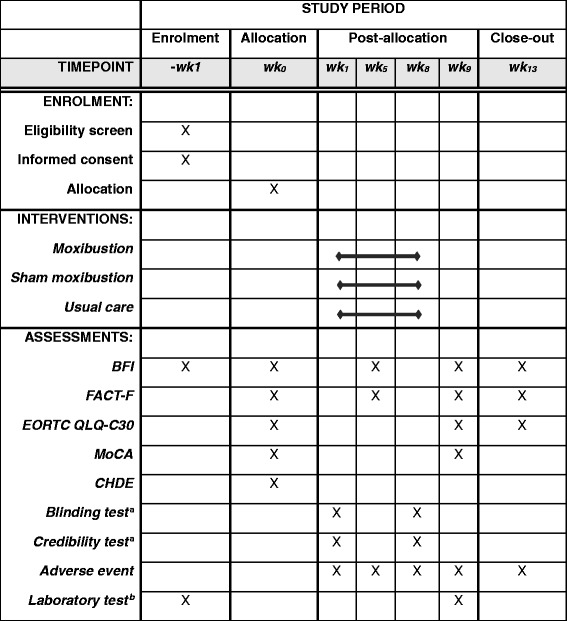
Schedule of enrolment, interventions, and assessments

*BFI* Brief Fatigue Inventory, *FACT-F* Functional Assessment of Cancer Therapy-Fatigue, *EORTC QLQ-C30* European Organization for Research and Treatment of Cancer Quality of Life Questionnaire, *MoCA* Montreal Cognitive Assessment, *CHDE* Questionnaire for Cold-Heat & Deficiency-Excess Patternization

^a^The blinding and credibility tests will be performed only for the moxibustion and sham moxibustion groups

^b^The laboratory test includes complete blood count and differential count, absolute neutrophil count, aspartate aminotransferase, alanine aminotransferase, total bilirubin, gamma-glutamyl transferase, blood urea nitrogen, creatinine, albumin, glucose, hemoglobin A1c, erythrocyte sedimentation rate, C-reactive protein, thyroid-stimulating hormone, free thyroxine, and human chorionic gonadotropin urine test (only for women in their childbearing years at the screening visit)

### Outcome measurements

The following outcome measures will be assessed by assessors blinded to the group allocations.

#### Primary outcome measure

The mean change in average BFI scores from baseline to the end of the 8-week intervention will be the primary outcome measure of the study. The BFI is a questionnaire composed of 9 questions related to the degree of fatigue and the level of daily life interference caused by the fatigue [33]. Zero to ten points are assigned to each question (0 = no fatigue or no interference; 10 = the most severe level of fatigue or complete interference). The level of the fatigue can be grouped into three categories according to the average BFI score: 1–3 (mild), 4–6 (moderate), 7–10 (severe) [33].

#### Secondary outcome measures

The secondary outcome measures will include changes in average BFI scores from baseline to week 5 and week 13 and changes in the Functional Assessment of Cancer Therapy-Fatigue (FACT-F) scores, European Organization for Research and Treatment of Cancer Quality of Life Questionnaire C-30 (EORTC QLQ) scores, and Montreal Cognitive Assessment (MoCA) scores from the baseline to weeks 5, 9, and 13.

### FACT-F

The Functional Assessment of Cancer Therapy-General (FACT-G) is an assessment tool used to evaluate various functions in the daily lives of cancer patients [34]. It is comprised of 27 items in 4 domains: physical well-being, social/family well-being, emotional well-being, and functional well-being. The FACT-F is a combined questionnaire of the FACT-G plus a fatigue subscale including 13 items focused on fatigue and its related symptoms [34]. Using the FACT-F, diverse aspects of the life and the fatigue of cancer patients can be comprehensively assessed.

### EORTC QLQ C-30

The EORTC QLQ C-30 was devised by the European Organization for Research and Treatment of Cancer (EORTC) to assess the QOL of cancer patients [35]. It includes 30 questions across the three domains of global health status/QOL, function scale and symptom scale. A variety of modules for specific cancer sites have been developed [35], although this study will adopt only the general version.

### MoCA

It is known that many patients undergoing cancer treatment suffer from cognitive decline, and fatigue can affect the cognitive function of cancer patients [36]. The MoCA will be used in this study to evaluate the level of cognition of patients. This assessment tool was developed to screen for mild cognitive impairment, a transitional phase characterized by the cognitive changes that occur between early and normal dementia [37]. In this study, the MoCA will be used to detect patients with mild cognitive impairment and to assess changes in cognitive function.

#### Safety outcomes

The participants will be instructed to voluntarily report all AEs that occurred during the trial. The investigators will determine whether the participants experienced any AEs via inquiries at each visit and checking for changes in laboratory tests from baseline to week 9. The laboratory tests will include complete blood count, differential count, absolute neutrophil count, aspartate aminotransferase, alanine aminotransferase, total bilirubin, gamma-glutamyl transferase, blood urea nitrogen, creatinine, albumin, glucose, hemoglobin A1c, erythrocyte sedimentation rate, CRP, thyroid-stimulating hormone, free thyroxine, and human chorionic gonadotropin urine test (only for women in their childbearing years at screening visit). All AEs identified during the trial will be recorded in the clinical record form.

The severity of AEs will be classified into five grades according to the common terminology criteria for AEs (CTCAE, version 4.0) developed by the National Cancer Institute as follows: mild, moderate, severe, life-threatening, or death [38]. In cases for which the CTCAE is not applicable, Spilker’s three level criteria will be used, and the AE will be classified into one of the three levels: mild, moderate, and severe [39].

A causal relationship between an AE and the moxibustion treatment provided in this study will be evaluated based on factors such as the temporal relationship, combined therapy, and underlying diseases, etc. The result of the causal relationship assessment will be categorized to one of the following six criteria: definitely related, probably related, possibly related, probably not related, definitely not related, or unknown.

#### Blinding test and credibility test

A blinding test will be conducted for the participants of the moxibustion group and the sham moxibustion group. Assessors who were not involved in the treatment procedure will administer the blind test questionnaire to the participants in these two groups after the first and last treatments of the trial. In the questionnaire, the participants will be asked to answer whether they think they had received real moxibustion, sham moxibustion, or that they had no idea. The result of this test will reveal whether patient blinding was successfully maintained in the trial.

Another questionnaire to evaluate the credibility of the intervention and the patient blinding will be provided to the participants of the two groups at the same time points. The credibility test is composed of four questions asking if the participant felt that the treatment could alleviate their fatigue, if they would recommend the treatment to a friend suffering from a similar complaint, if the treatment seems logical to them, and if the treatment could successfully alleviate other complaints. The result of the test will show the participants’ expectations and confidence in the moxibustion treatment performed in this trial ranging from 0 (very low confidence) to 6 (very high confidence) [40].

### Data monitoring and quality control

Data collection will be performed in accordance with the pre-approved protocols, and the quality of the study will be managed by regular monitoring. The monitoring will evaluate whether the recruitment and intervention procedures followed the protocol and that the record of the case report form matches the original document.

To ensure the quality of the study, all researchers should complete a good clinical practice training course. To maintain consistency of the procedures among research centers, we will provide detailed information of every step of this trial to all researchers at the researcher meetings and maintain communication with all researchers throughout the study period. The practitioners will be limited to those who have been licensed as a doctor of Korean medicine by the Ministry of Health and Welfare of South Korea and have a clinical career of 2 or more years.

### Sample size calculation

The primary outcome of this study will be the mean change in average BFI scores from baseline to week 9. Based on a previous study [41], we estimated that the mean difference of the pre- and post-treatment change in the average BFI scores between the moxibustion and sham moxibustion group will be −1.6 and the standard deviation will be 2.0. With a 5% significance level and 80% power, the sample size was calculated to be 25. Considering a 20% drop-out rate, 32 participants are required per group. In conclusion, a total of 96 participants are needed in this trial.

### Statistical analysis

An independent statistician blinded to group allocation will perform statistical analysis using SAS® Version 9.4 (SAS institute. Inc., Cary, NC). All statistical analyses will be two-tailed, and the significance level will be set at 5%.

Three types of analysis sets will be used in this study, as follows: intention to treat (ITT) analysis set, full analysis set (FAS), and per-protocol (PP) set. The ITT analysis set will be comprised of the data from every participant who had been randomly assigned to any group in this trial. The FAS set will include the data from all participants who ever received the intervention and provided assessment data at least once following random allocation. The PP set will include only the data from participants who completed the trial without any major violation of the protocol. The FAS will be the primary set for the efficacy analysis, and sensitivity analysis will be conducted to compare the results from the FAS and PP sets. For safety analysis, the ITT set will be the primary set, and the PP set will be used as a supplement. The multiple imputation method will be adopted to handle missing data in incomplete data sets.

Summary statistics of demographic characteristics and baseline measurements of the variables of each group will be presented. Continuous data will be expressed as the mean ± standard deviation, but median and 95% confidence intervals will be provided if necessary. Between-group differences will be analyzed using analysis of variance (ANOVA) or the Kruskal-Wallis test. Categorical data will be presented as frequencies with percentiles and analyzed using the Chi-square test or the Fisher’s exact test.

The primary outcome measure of this study will be the mean change of average BFI scores acquired at baseline and week 9. To validate significant changes in BFI between groups, we assumed two sets of hypotheses.

**The null hypothesis (H**_**01**_ **):** There is no difference in the mean change of the BFI before and after treatment between the moxibustion group and the usual care group.

**The alternative hypothesis (H**_**11**_ **):** There is a difference in the mean change of the BFI before and after treatment between the moxibustion group and the usual care group.

**The null hypothesis (H**_**02**_ **):** There is no difference in the mean change of the BFI before and after treatment between the moxibustion group and the sham moxibustion group.

**The alternative hypothesis (H**_**12**_ **):** There is a difference in the mean change of the BFI before and after treatment between the moxibustion group and the sham moxibustion group.

To test the two hypotheses, analysis of covariance (ANCOVA) will be utilized with the baseline BFI value as a covariate and each group as a fixed factor. If any clinically significant difference between groups is identified among the demographic characteristics or the variables measured at baseline, it will be adjusted based on its covariance. The problem of multiple comparison will be solved using the fixed sequence method. In other words, if the H_01_ is rejected and H_11_ is adopted in the statistical test for the first hypothesis set, and then the test for H_02_ will performed. However, when the H_01_ is adoptted, the second hypothesis set for H_02_ will not be verified [42].

The secondary outcome measures of the study will be the mean changes in the BFI, FACT-F, EORTC QLQ C-30 and MoCA before and after the interventions in the three groups. They will be analyzed via the same methods used for the primary outcome measure analysis. Student’s paired t-tests or Wilcoxon signed-rank tests will be used to analyze the differences in the measured values before and after treatment in each group. ANOVA with Dunnett’s post hoc test will be performed to compare the differences in trends per visit. Subgroup analyses will be conducted to check whether there are significant differences in the responses to moxibustion treatment depending on the severity of fatigue, the expected level of treatment, or patterns of cold-heat and deficiency-excess measured at baseline.

## Discussion

The purpose of this study is to assess the efficacy and safety of moxibustion treatment for patients with cancer-related fatigue.

Cancer-related fatigue is one of the most prominent manifestations of cancer survivors that can be occur over a long period of time even after the end of cancer treatment or the complete remission of cancer [3]. Although the prevalence of cancer-related fatigue varies among studies, it is generally reported that approximately 60 to 100% of cancer survivors experience cancer-related fatigue [43]. Cancer-related fatigue lowers the QOL of the family of the patient in addition to the cancer patient and can become a socially obvious burden [5–7]. Although a variety of pharmacologic and nonpharmacologic therapies for treating cancer-related fatigue have been evaluated in the clinical trials, no treatments have been confirmed as valid and safe for long-term use [17, 19]. Therefore, it is important to confirm whether moxibustion, one of the oldest but most popular therapies in traditional East Asian medicine, including Korean medicine, is a viable therapeutic option for the management of cancer-related fatigue.

The moxibustion treatment regimen was established based on the findings of the relative studies that were previously published. Although there were few studies with good quality evaluating the effect of moxibustion for the patients with cancer-related fatigue, a recent publication by Mao et al. [32]. using infrared laser moxibustion was helpful. Mao et al. [32]. reported that the moxibustion reduced the BFI score during the 4-week treatment period, however, it tended to stagnate at follow-up session at week 8. Another study by Mao et al. [44] targeting the breast cancer patients with psychological distress including cancer-related fatigue was also reliable even though its intervention was electroacupuncture, not moxibustion. According to this study, the BFI score decreased during the 8-week of treatment period, and then the improvement trend was stopped 4 weeks after the completion of the treatment. Therefore, we expect to see how the BFI will change during and after the 8-week treatment period.

It is known that the placebo effect has a high proportion in the palliative care for the cancer survivors with cancer-related fatigue [45]. It varies by 27–60% depending on the studies [45]. Besides, the sham control interventions of traditional medicine such as moxibustion or acupuncture are not reported to be physiologically inert [46, 47]. Considering the clinical characteristics of the cancer-related fatigue which is sensitive to the placebo response and the unique nature of the sham moxibustion, we cannot rule out the possibility that the difference between the effects of real moxibustion and sham moxibustion will not be statistically significant even though the moxibustion treatment is actually effective because of the placebo effect. This is why we designed a 3-arm parallel study of the usual care group as well as the sham control group.

There are some limitations to this study. First, the outcome measures do not include objective indicators for assessing the level of fatigue. Because fatigue itself is defined as the subjective feeling of the patient, there is currently no way to objectively evaluate it. Some studies have reported that various biomarkers, such as TNF-alpha, interleukin (IL)-1 beta, IL-6, IL-2, CRP, etc., are associated with cancer-related fatigue [6, 10], although the results of these studies are not consistent in regarding this issue. Therefore, there is currently no confirmed biomarker that represents the level of cancer-related fatigue [48]. When definitive biomarkers are discovered in the future, additional clinical trials will need to consider them. Nevertheless, the use of questionnaires to regarding the level of the subjective feelings of fatigue of the subjects is the most widely used method to assess or quantify fatigue as objectively as possible in the field, and we adopted the BFI and FACT-F according to this gold-standard.

Second, there is a limitation pertaining to incomplete blinding. We used moxibustion devices that appeared to be identical in the moxibustion and sham moxibustion groups in an attempt to achieve patient blinding between these two groups. However, it is not possible to blind the participants in the usual care group that do not receive moxibustion treatment. Thus, there could be a concern that this incomplete blinding of participants in the usual care group may introduce bias into the results regarding the outcome measures based on the subjective symptoms that the patients feel.

However, Atkinson et al. [49] recently showed that there is no clear evidence that the patient blinding is a source of bias in the patient-reported outcome (PRO) or at least that the bias significantly affects the results of the PRO. They insisted that, in clinical trials of therapies for cancer patients, the use of PRO, which is essential for directly assessing the risks, benefits, and value of the therapies to cancer patients, should be promoted regardless of patient blinding because there is no other means that provides information by completely capturing the patient’s experience related to the treatment [49]. Based on such expert opinions, we will adopt a three-armed parallel design, including a usual care group in addition to a sham control group, in this trial even though the primary outcome measure is highly dependent on the patients’ subjectivity.

There is another limitation in terms of patient blinding. The moxibustion delivered to the sham group is designed to not deliver complete warmth to the site of application; therefore, the patients of this group may notice that the treatment is not real moxibustion because it is not sufficiently hot. To prevent this possibility, only patients who have never received moxibustion treatment should be recruited for inclusion. However, this inclusion criterion could significantly lower the recruitment rate. Thus, we decided to register patients regardless of their past experience using moxibustion treatment. To further mitigate this limitation, the real and sham moxibustion devices used in the study will have the same appearance. In addition, the patients will be allowed observe the procedures that the practitioner carries out while operating the moxibustion devices: In case of IM, the participants of both groups will see the smoke and smell the odor from the burning mugwort moxa cone after the practitioner ignites it; and in case of EM, the participants will see the practitioner turn on the EM, which will begin to blink. Finally, we will perform a blinding test to confirm whether patient blinding had been successfully achieved in these two groups.

There are a variety of types in cancer, although patients with cancer-related fatigue will be allowed to participate in this study regardless of cancer type. Some researchers have suggested that patients with cancer at specific sites may be more vulnerable to cancer-related fatigue [14], although it has not been clearly investigated how specific types of cancer develop or affect fatigue. Therefore, we will comprehensively recruit patients regardless of cancer type in this study. However, if the results of this study or future studies by experts in the field demonstrate more promising clues regarding the relationship between specific cancer types and cancer-related fatigue, a new clinical trial targeting only patients with limited types of cancer should be designed.

Cancer-related fatigue is a symptom that patients can experience before cancer diagnosis, during cancer progression, during cancer treatment, after cancer treatment, or even after cancer remission. However, ‘during cancer treatment’ and ‘immediately after cancer treatment’ are known time periods during which cancer-related fatigue is most common and most prominent [6]. It has also been reported that the type of cancer treatment such as chemotherapy or radiotherapy can contribute in different ways to cancer-related fatigue [50]. The regimen of the treatment, for example, the type of drugs or the dose of radiation, might differentially affect fatigue. However, considering the practical feasibility of the clinical trial, we decided to exclude those who are currently undergoing active treatment for cancer or scheduled to receive cancer treatment in the near future. Therefore, the results of this study will not shed light on the effect of moxibustion at the peak of cancer-related fatigue.

One of the distinct features of this study is that the participants will be categorized based on patterns as cold or hot and as deficiency or excess. In traditional medicine, pattern identification is a way to categorize a patient according to the signs and symptoms of several patterns, and doctors can determine how to treat the patient based on the identified patterns. Moxibustion is a widely used intervention in traditional Korean medicine and is often chosen as a better option, especially for patients categorized by cold or deficiency patterns. For this reason, we will adopt the questionnaire for cold-hot and deficiency-excess pattern categorization [51]. We will conduct subgroup analyses according to the identified patterns to determine whether moxibustion is more effective in cold-type patients than in hot-type patients and whether it improves the symptoms of deficiency-type patients better than excess-type patients. We will also determine whether such patterns are similar or different between the sham moxibustion group and the usual care group. We believe that such an analysis is a meaningful scientific exploration for investigating the clinical and practical value of the concept in traditional Korean medical theory, and the results of this study will help to identify patients who are best suited for moxibustion and related therapies.

Another characteristic of this study is that it assesses not only fatigue but other biobehavioral factors, such as QOL and cognitive function. A previous study reported that cancer-related fatigue negatively influences the QOL and cognitive function of patients [52]. Thus, we seek to comprehensively evaluate the effect of moxibustion on various aspects of daily life that patients with cancer-related fatigue experience.

A variety of factors are known to influence the cancer-related fatigue as well as cancer treatment and cancer itself [11]. In this study, the patients with prominent risk factors that are known to contribute to cancer-related fatigue will be screened out to eliminate confounding factors and to focus on cancer-related fatigue. Thus, the eligibility criteria include the exclusion of patients who have current severe inflammation or anemia, clinically significant insomnia, moderate or higher levels of emotional disturbances such as anxiety or depression, and definite cancer pain. The attempt to select purely tired cancer patients with minimal other pathological causes of fatigue is a strength of this study, although as a result, the results of this study cannot be applied to patients with cancer-related fatigue combined with these conditions. Therefore, these results as they apply to patients with multiple conditions need to be further examined in future studies.

### Trial status

The trial is currently recruiting participants.

## Additional files

Additional file 1: Details of moxibustion and sham moxibustion treatments based on the Standards for Reporting Interventions in Clinical Trials of Acupuncture (STRICTA) Checklist. (DOCX 20 kb)

Additional file 2: Standard Protocol Items: Recommendations for Interventional Trials (SPIRIT) checklist. (DOC 123 kb)

## Abbreviations

AE: Adverse event
ANCOVA: Analysis of covariance
ANOVA: Analysis of variance
BFI: Brief Fatigue Inventory
CAM: Complementary alternative medicine
CONSORT: Consolidated Standards of Reporting Trials
CRP: C-reactive protein
CTCAE: Common terminology criteria for adverse events
ECOG: The Eastern Cooperative Oncology Group
EM: Electrical moxibustion apparatus
EORTC QLQ C-30: European Organization for Research and Treatment of Cancer Quality of Life Questionnaire
EORTC: European Organization for Research and Treatment of Cancer
FACT-F: Functional Assessment of Cancer Therapy-Fatigue
FACT-G: Functional Assessment of Cancer Therapy-General
HADS: Hospital Anxiety and Depression Scale
HPA: Hypothalamic-pituitary-adrenal
ICD-10-CM: International Classification of Diseases, tenth revision clinical modification
IL: Interleukin
IM: Ignition-type moxibustion device
ISI: Insomnia severity index
MoCA: Montreal Cognitive Assessment
NRS: Numeric rating scale
PRO: Patient-reported outcome
QOL: Quality of life
SAE: Serious adverse reaction
STRICTA: Standards for Reporting Interventions in Clinical Trials of Acupuncture
TNF: Tumor necrosis factor
