# Supplementary material for: Moxibustion for cancer-related fatigue: study protocol for a randomized controlled trial
Source: BMC Complement Altern Med. 2017 Jul 5;17:353. doi: 10.1186/s12906-017-1856-3 (PMC5499061; doi:10.1186/s12906-017-1856-3)
Supplement: Supplementary file 1 — Details of moxibustion and sham moxibustion treatments based on the Standards for Reporting Interventions in Clinical Trials of Acupuncture (STRICTA) Checklist. (DOCX 20 kb) [file 12906_2017_1856_MOESM1_ESM.docx]

**Additional file 1** Details of moxibustion and sham moxibustion treatment based on the Standards for Reporting Interventions in Clinical Trials of Acupuncture (STRICTA) Checklist

| **Item** | **Detail** | **Description** |
| --- | --- | --- |
| **1. Acupuncture**  **rationale** | 1a) Style of acupuncture [style of moxibustion] | Moxibustion based on traditional Korea medicine |
|  | 1b) Reasoning for treatment provided, based on historical context, literature sources, and/or consensus methods, with references where appropriate | Textbook of Acupuncture and Moxibustion Medicine in Korean medicine [24], related papers [26,31,32], and expert (doctors of Korean medicine) consensus |
|  | 1c) Extent to which treatment was varied | Standardized treatment |
| **2. Details of needling** | 2a) Number of needle insertions per subject per session [number of acupoints used] | 4 (number of acupoints used per subject per session) |
|  | 2b) Names of points used | CV2, CV8, LI4 (bilateral), ST36 (bilateral) |
|  | 2c) Depth of insertion, based on a specified unit of measurement, or on a particular tissue level | Not applicable |
|  | 2d) Response sought | Sense of warmth |
|  | 2e) Needle stimulation [type of moxibustion devices used in the trial] | 1) Ignition type moxibustion device: at CV2, CV8  2) Electrical moxibustion apparatus: at LI4, ST36 |
|  | 2f) Needle retention time [moxibustion retention time] | 30 minutes |
|  | 2g) Needle type (diameter, length, and manufacturer or material) [Details of the moxibustion materials] | A mugwort cone (diameter 2.2 cm, height 2.2 cm, Brand name: *Hatnim* smokelss moxa cone, manufacturer: *Bosung*) in an ignition type moxibustion device (diameter 5.8 cm, height 4.0 cm, Brand name: *Hatnim Shingigu*);  A Cube-shaped electrical moxibustion apparatus (20 x 28 x 18 mm, Brand name: *Cettum*, manufacturer: Foretek Microsystem Co., Ltd.) |
| **3. Treatment regimen** | 3a) Number of treatment sessions | 16 |
|  | 3b) Frequency and duration of treatment sessions | Once a day, twice a week for 8 weeks |
| **4. Other components of treatment** | 4a) Details of other interventions administered to the acupuncture group [details of other interventions administered to the moxibustion group] | - No additional Korean traditional medical treatment regarding cancer-related fatigue is allowed except for what is given for the study.  - The interventions other than Korean traditional medical treatment to mitigate cancer-related fatigue that have been steadily received for more than 4 weeks before the screening visit are allowed to continue during the study, but no additional treatment is allowed after the beginning of the trial.  - All the three groups will be educated with a brochure about the cancer-related fatigue. |
|  | 4b) Setting and context of treatment, including instructions to practitioners, and information and explanations to patients | - Clinical trial centers in university hospitals  - Participants will be informed that moxibustion treatment for cancer-related fatigue will be perforemd based on traditional Korean medicine  and unnecessary conversion will be limited during the procedure. |
| **5. Practitioner background** | 5) Description of participating acupuncturists (qualification or professional affiliation, years in acupuncture practice, other relevant experience) [description of practitioners] | Licensed Korean medical doctors with at least 2 years of clinical experience and 6 years of Korean medicine college education will perform the moxibustion procedure. |
| **6. Control or comparator interventions** | 6a) Rationale for the control or comparator in the context of the research question, with sources that justify this choice | A previous research showing the reliability of sham moxibustion that blocks the heat channel [26]. |
|  | 6b) Precise description of the control or comparator. If sham acupuncture or any other type of acupuncture-like control is used, provide details as for Items 1 to 3 above. [description of sham moxibustion] | 1a. sham device for ignition type moxibustion: the moxibustion devices of which the channel on the bases are blocked by insulator will be applied on abdomen  1b. sham apparatus for electrical moxibustion: the moxibustion apparatuses of which the heat sensor in the electrical board of the base is controlled to block heat transfer will be attached on four limbs.  2. The locations of points that will be used:  Abdomen (about 13.5 cm above the bilateral 3 cm points from umbilicus), Upper limb (1 cm lateral and 5 cm distal points from cubital creases of bilateral arms), Lower limb (Upper 1/3 points of medial line of bilateral tibia)  3. Treatment duration, frequency, period: the same as the treatment group |
